# Case Report: Novel Mutations in the *PCCB* Gene Causing Late-Onset Propionic Acidemia

**DOI:** 10.3389/fgene.2022.807822

**Published:** 2022-03-17

**Authors:** Guang Ji, Yaling Liu, Xueqin Song, Zhenfei Li

**Affiliations:** Department of Neurology, Second Hospital of Hebei Medical University, Shijiazhuang, China

**Keywords:** PCCB gene, novel mutation, late onset, propionic acidemia, clinical exome sequencing

## Abstract

**Introduction:** Propionic acidemia is an autosomal recessive metabolic disorder and the patients with adult onset are very rare.

**Methods:** Two PCCB mutations were identified. Clinical data were collected from a patient, and metabolic screening and clinical exome sequencing analysis were performed.

**Results:** Two novel mutations were identified in the PCCB gene: M1:c.404_406del:p.G135del and M2:c.632C>T:p.T211I.

**Conclusion:** Late-onset propionic acidemia should be taken into account, and metabolic screening as well as gene analysis should be performed to make a definite diagnosis timely.

## Introduction

Propionic acidemia (PA) is an autosomal recessive disorder of organic acid metabolism caused by the accumulation of toxic metabolites. It is due to the deficiency of the mitochondrial enzyme propionyl-CoA carboxylase (PCC), which catalyzes the carboxylation of propionyl-CoA to methylmalonyl-CoA (Wongkittichote et al., 2017). PCC is an α6β6 multimer composed of α and β subunits, encoded by the genes of *PCCA* and *PCCB*, respectively (Campeau et al., 2001). The main function of PCC is to accelerate the conversion of propionyl-CoA to methylmalonyl-CoA.

According to the time of onset, PA can be divided into neonatal onset and late onset. The clinical manifestations are varied and lack of specificity. Neonatal-onset PA is within 1 year old. It is normal at birth and occurs a few days later. The initial symptoms are feeding difficulties, vomiting, dehydration, hypothermia, lethargy, hypotonia, convulsion, and dyspnea. If not treated in time, the condition will aggravate, with ketosis, metabolic acidosis, hyperammonemia, and coma.

Individuals with late-onset PA exhibit significant differences in clinical manifestations, which can occur from 1-year old to adulthood. The common early manifestations are anorexia, growth retardation, convulsion, hypotonia, and abnormal mental behavior. The acute metabolic crisis is often induced by fever, hunger, a high protein diet, and infection. The accumulation of organic acid metabolites, such as propionic acid, often causes bone marrow suppression, anemia, granulocytopenia, and thrombocytopenia, prone to infection and bleeding. Very few patients can also show optic nerve atrophy, hearing loss, chronic renal failure, and premature ovarian failure.

Here we report a patient with PA who was screened by tandem mass spectrometry (MS/MS) and urine gas chromatography MS. Using clinical exome sequencing (CES) analysis, two novel mutations in the *PCCB* gene were found in the patient.

## Case Report

The patient was the only child in the family. The birth and development of the patient were normal. He felt tired in his lower limbs with lumbago after a long-distance walk from the age of 22 years (Table 1). After a lot of exercises or a high-protein-diet intake, he developed nausea, vomiting, and, in severe cases, unconsciousness. On physical examination, the patient was alert and oriented, and no obvious abnormality was found in the nervous system examination.

**TABLE 1:** The timeline with relevant data from the episode of care.

Time	Episode of care	Treatment and prognosis
2015.12	First onset	Protein-restricted diet and treatment of reducing ammonia; improvement of symptoms
2019.6	Second onset	Reducing-ammonia treatment; symptoms improved again
2019.10	Third onset	Cardiotonic and diuretic treatment; symptoms worsened
2019.11	Metabolic acidosis	Correct acidosis; dead

**FIGURE 1:**
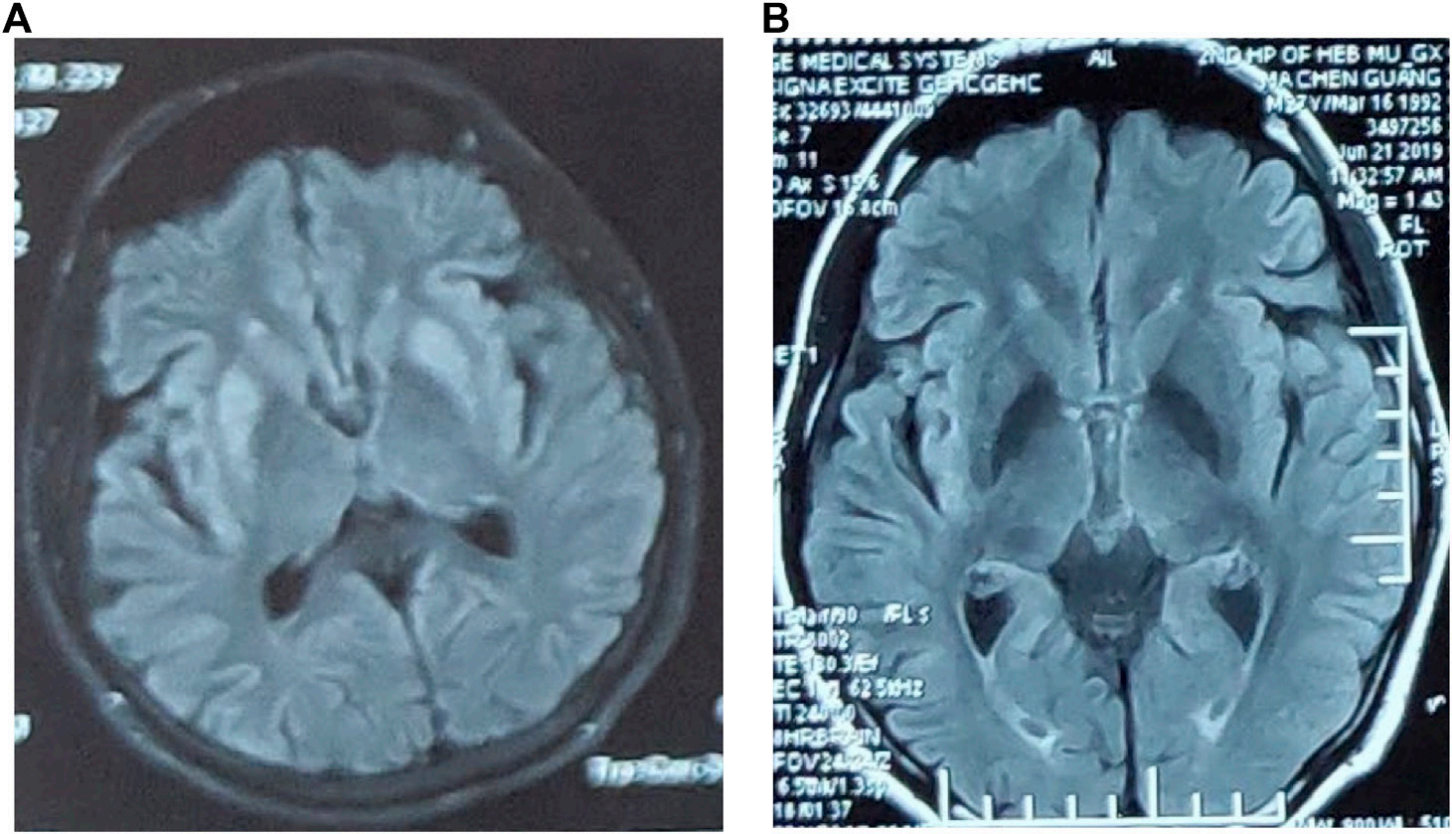
**(A)** Brain MRI showed symmetrical abnormal signals in bilateral basal ganglia. **(B)** The abnormal signal in the bilateral basal ganglia in the brain MRI disappeared.

The results of liver function showed an increased level of blood ammonia (98.9 µmol/L; normal range, 6–35 µmol/L) and a decreased level of ceruloplasmin (0.144 µmol/L; normal range, 6–35 µmol/L). Cerebrospinal fluid analysis showed normal cell count of 1/mm^3^ (normal <5/mm^3^), a decreased glucose of 3 mmol/L (normal range, 3.6–4.5 mmol/L), and a normal total protein of 35 mg/dL (normal value, <45 mg/dL). Brain magnetic resonance imaging (MRI) showed symmetrical abnormal signals in bilateral basal ganglia (Figure 1A). Electromyogram was normal.

After a protein-restricted diet and treatment of reducing ammonia, the symptoms of weakness in lower limbs and lumbago were once relieved. However, at the age of 26 years, he again developed nausea, vomiting, and diarrhea, followed by numbness and weakness of both lower limbs and transient unconsciousness in severe cases when walking (Table 1). The blood ammonia increased to 108 µmol/L, whereas the abnormal signal in bilateral basal ganglia of brain MRI disappeared (Figure 1B). The symptoms improved again after reducing-ammonia treatment. Four months later (Table 1), the patient developed chest tightness, dyspnea, and general fatigue. The blood gas analysis showed pH 7.438, Pco_2_ 26.2 mm Hg, and Po_2_ 105.2 mm Hg. Increased level of troponin (0.51 ng/mL) and brain natriuretic peptide (266 ng/mL) was also detected. Echocardiography showed noncompaction of the myocardium and normal left ventricular systolic function. After admission, the patient’s symptoms gradually worsened with unconsciousness and irritability. The blood gas analysis upon admission showed pH 7.007, Pco_2_ 22.6 mm Hg, and lactate 20.05 mmol/L, which indicated metabolic acidosis. After correction of acidosis and ventilator-assisted respiration, the patient did not respond to the treatment and died at last (Table 1).

The level of propionyl carnitine was 8.71 µM (normal range, 0.30–5.00 µM). The ratio of propionyl carnitine to acetyl carnitine was 0.33 (normal range, 0.02–0.20). The increase in these indicators suggested methylmalonic acidemia or PA. By urine organic acid analysis, elevated indicators of hydroxypropionate 23.8 (normal range, 0–4.0 (μ)mol/mol creatinine), propionyl glycine 7.8 (normal range, 0–0.4 (μ)mol/mol creatinine), and methyl citrate 33.9 (normal range, 0.0–0.7 (μ)mol/mol creatinine) were detected, whereas methylmalonic acid (0.0 (μ)mol/mol creatinine) was not present in the urine sample. The above results indicated the diagnosis of PA.

### Clinical Exome Sequencing

Peripheral blood was obtained from the patient and his parents. DNA was isolated from peripheral leukocytes using a commercial kit (TIANGEN, China). The quantity/quality of DNA was assessed using Onedrop OD1000 spectrophotometer and by agarose gel electrophoresis. CES analysis was done in Shanghai We-Health Biomedical Technology Co., Ltd.

Two heterozygous mutations of *PCCB* were identified in the patient. According to the ACMG standards and guidelines for the interpretation of sequence variants, the clinical significance of these two mutations was still uncertain. The two *PCCB* mutations were confirmed by Sanger sequencing.

**FIGURE 2:**
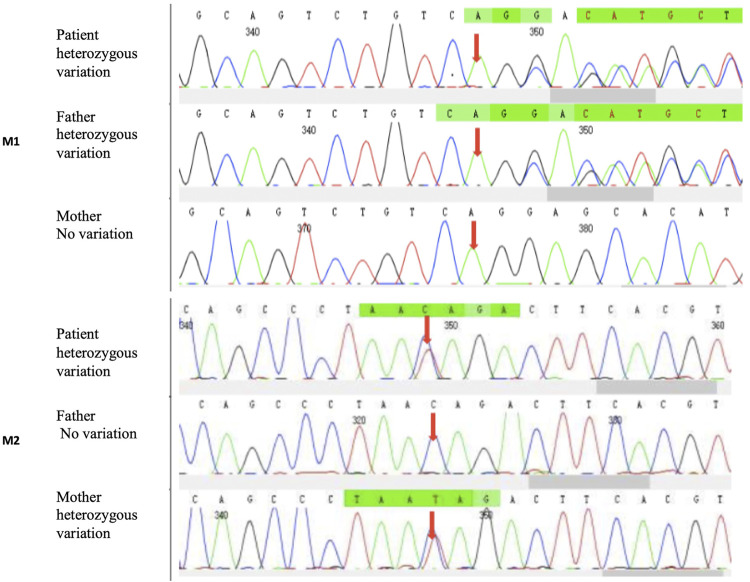
CES identified one heterozygous deletion mutation. M1: c.404_406del:p.G135del and one heterozygous missense mutation. M2: c.632C>T:p.T211I. Sanger sequencing results showed that the mutations M1 and M2 were inherited from the father and mother, respectively.

CES identified one heterozygous deletion mutation M1: c.404_406del:p.G135del, which resulted in the deletion of amino acid at position 153 in the *PCCB* gene but did not cause frameshift. Another missense mutation M2: c.632C>T:p.T211I, resulted in the change of codon 211 in the *PCCB* gene from threonine to isoleucine. Both mutations are novel. Sanger sequencing results confirmed that the mutations M1 and M2 were inherited from the father and mother, respectively (Figure 2). The results above indicated that the proband’s healthy parents were heterozygous carriers. The abnormality of the *PCCB* gene can lead to autosomal recessive PA.

## Discussion

The dysfunctions of PCC cause propionyl-CoA accumulation in the body and produce a large number of abnormal metabolites (propionic acid, 3-hydroxypropionate, and methyl citrate) by activating the bypass metabolic pathway. Abnormal metabolites can produce a series of clinical symptoms, including feeding difficulties, vomiting, lethargy, coma, metabolic acidosis, and hyperammonemia.

According to the occurrence of the first symptoms, PA can be divided into early-onset (first symptoms occurred within 28 days of life) and late-onset (symptoms started after the neonatal period). Most PA patients develop symptoms within 1 year of age (Lehnert et al., 1994; Van der Meer et al., 1996). Very few patients have the first symptom after 1-year of age (Nyhan et al., 1999; Perezcerda, 2004). In this case, the first symptoms appeared at the age of 22 years.

The clinical and biochemical picture of late-onset PA is more heterogeneous. Clinical findings, neuroimaging, and neuropathology show a high frequency of basal ganglia lesions in late-onset PA patients (Hamilton et al., 1995; Bergman et al., 1996). While the pathogenesis of the basal ganglia abnormalities is not clear, a possible explanation is that the basal ganglia region is of high energy metabolism, which is more vulnerable to disease than other regions. Hyperammonemia may be one of the causes of basal ganglia damage. Studies have found that organic acids and hyperammonemia can cause brain damage in rats, including vacuolization, ischemic neurons, and pericellular edema (Viegas et al., 2014). In this case, the abnormal signals in bilateral basal ganglia of brain MRI disappeared after he received reducing-ammonia treatment, which also suggests the close relations between hyperammonemia and the damage of basal ganglia.

Another typical clinical manifestation of the patient was fatigue intolerance. When the patient walked 100 m, he felt tired and needed a rest. This feature is similar to the manifestation of metabolic myopathy. Some studies have found that patients with PA had impaired lipolysis, blunted fatty acid oxidation (FAO), a compensatory increase in carbohydrate utilization, and low work capacity (Storgaard et al., 2020). In normal people, the level of palmitate and free fatty acid (FFA) increased after exercise, but in PA patients, the increase in palmitate and FFA was not obvious. Impaired lipolysis, lack of substrates for FAO, low plasma carnitine concentration, and impaired TCA cycle are most likely to cause the blunted increase in palmitate oxidation and total FAO. As a result, PA patients rely more on carbohydrates as an energy source than healthy people. Similarly, the low work capacity in PA patients may also be ascribed to a shortage of substrates in the TCA cycle. These findings are similar to those reported in a patient with neutral lipid storage disease, which suggests that PA should be added to the list of metabolic myopathies (Laforêt et al., 2012).

PA is a serious, life-threatening metabolic disorder, and several related complications have been described in cases published before. Cardiac complications have been observed in PA including cardiomyopathy and arrhythmias. Cardiomyopathy, dilated usually, has been described in several PA patients, and the age at onset ranged between 4 weeks of age and adulthood, although a portion of the patients were diagnosed during a metabolic crisis (Pena et al., 2012). Echocardiography did not find dilated cardiomyopathy in this patient but showed noncompaction myocardium. Unfortunately, before the results of the metabolic screening and CES analysis, the patient developed cardiac shock during hospitalization and died of circulatory failure within a few days, which may be caused by the myocardial damage involved in the metabolic crisis.

We found two novel compound heterozygous mutations in the PCCB gene causing late-onset PA, including a deletion mutation, which resulted in the deletion of amino acid at position 153 of the *PCCB* gene, but did not shift the frame, and a missense mutation in the *PCCB* gene, which resulted in the change of codon 211 of the *PCCB* gene from threonine to isoleucine. These two novel mutations may be related to mild impairment of PCC function so that clinical symptoms occurred late. Most affected individuals of PA are compound heterozygotes (Pena et al., 2012). The majority of these mutations were missense mutations (40%), followed by small insertions/deletions and splicing mutations. There is a strong relationship between the genotype and phenotype observed in many cases of PA; some were found to affect the protein function and were associated with an early onset and severe clinical phenotype; on the other hand, some were found with mild phenotypes (Zayed, 2015). However, the fact that compound heterozygosity is present in the majority of patients makes it difficult to establish genotype–phenotype correlations; furthermore, population-specific mutations may exist, and it is hard to predict their effect on other ethnic groups with different genetic makeup (Zayed, 2015).

PA at adult onset is very rare. If the patient presents with fatigue intolerance, disturbance of consciousness, hypotonia, seizures, ataxia, vomiting, neutropenia, and thrombocytopenia, this disease should be taken into consideration. Screening of organic acid metabolism in blood and urine as well as gene analysis should be performed to make a definite diagnosis timely.

Statement: The appropriate intervention was not possible as the patient died before the diagnosis could be completed.

## Data Availability

The datasets for this article are not publicly available due to concerns regarding participant/patient anonymity. Requests to access the datasets should be directed to the corresponding author.
